# Measuring Knee Bone Marrow Perfusion Using Arterial Spin Labeling at 3 T

**DOI:** 10.1038/s41598-020-62110-y

**Published:** 2020-03-24

**Authors:** Xiufeng Li, Casey P. Johnson, Jutta Ellermann

**Affiliations:** 1 Center for Magnetic Resonance Research, University of Minnesota, Minneapolis, MN USA; 2 Veterinary Clinical Sciences Department, University of Minnesota, Saint Paul, MN USA

**Keywords:** Translational research, Bone

## Abstract

Bone perfusion is an essential physiological measure reflecting vasculature status and tissue viability of the skeletal system. Arterial spin labeling (ASL), as a non-invasive and non-contrast enhanced perfusion imaging method, is an attractive approach for human research studies. To evaluate the feasibility of ASL perfusion imaging of knee bone marrow in the distal femoral condyle at a 3 T MRI scanner, a study was performed with eight healthy volunteers (three males and five females, 26 ± 2 years old) and two patients (male, 15 and 11 years old) with diagnosed stage II juvenile osteochondritis dissecans (JOCD). ASL imaging utilized a flow-sensitive alternating inversion recovery method for labeling and a single-shot fast spin echo sequence for image readout. In addition to quantitative knee bone marrow ASL imaging, studies were also performed to evaluate the effects of prolonged post-bolus delay and varied labeling size. ASL imaging was successfully performed with all volunteers. Despite the benefits of hyper-intensive signal suppression within bone marrow, the use of a prolonged post-bolus delay caused excessive perfusion signal decay, resulting in low perfusion signal-to-noise ratio (SNR) and poor image quality. Bone marrow perfusion signal changed with the labeling size, suggesting that the measured bone marrow perfusion signal is flow-associated. The means and standard deviations of bone marrow blood flow, spatial SNR, and temporal SNR from the quantitative perfusion study were 38.3 ± 5.2 mL/100 g/min, 3.31 ± 0.48, and 1.33 ± 0.31, respectively. The imaging results from JOCD patients demonstrated the potential of ASL imaging to detect disease-associated bone marrow perfusion changes. This study demonstrates that it is feasible to perform ASL imaging of knee bone marrow in the distal femoral condyle at 3 T.

## Introduction

Perfusion (or blood flow) is an essential physiological measure reflecting vasculature status and tissue viability. As in other tissues and organs, bone perfusion also plays a pivotal role in maintaining healthy status, preventing disease initiation and development, sustaining post-injury healing and regrowth, and facilitating therapeutic drug delivery for stimulated healing^1–3^. Imaging and monitoring bone marrow perfusion changes can assist the management of developmental skeletal diseases, such as chronic osteoarthritis^4^ and juvenile osteochondritis dissecans (JOCD)^5^.

*In vivo* evaluation of bone perfusion can be achieved by using different imaging modalities, such as positron emission tomography (PET) with 18F-FDG or dynamic contrast-enhanced (DCE) magnetic resonance imaging (MRI)^6–13^. Although DCE MRI has been applied to measure bone marrow perfusion, the health risks of MRI with gadolinium-based contrast agents (e.g. nephrogenic systemic fibrosis)^14^ have raised significant concerns for its routine applications^15^. In contrast to these imaging methods, arterial spin labeling (ASL) imaging utilizes endogenous blood water as an intrinsic agent to measure tissue perfusion^16^. As a non-radioactive, non-contrast-enhanced and non-invasive approach, ASL imaging eliminates the concerns on the exposure of ionizing radiation or the potential health risks of gadolinium-based contrast agents when they are applied for human research studies. Thus, ASL is well suited for longitudinal monitoring of disease progression and routine evaluation of therapy response, particularly in patients with renal deficiency and in children.

However, ASL imaging of the skeletal system is challenging: (1) ASL imaging is a low signal-to-noise ratio (SNR) technique, and bone perfusion level is lower than those in highly perfused organs, such as the brain and kidneys^17^; and (2) complicated vascular structure of blood feeding arteries for the skeletal system and low blood flow velocities^18,19^ make it difficult to utilize high SNR ASL imaging methods, such as pseudo-continuous ASL^20^. Recently, the feasibility of measuring bone marrow perfusion using ASL imaging was demonstrated in the vertebrae at 3 T^21^, and also explored in the knee at 7 T^22^.

The purpose of this study was to investigate the feasibility of knee bone marrow perfusion imaging using a pulsed ASL method at 3 T.

## Results

**Figure 1:**
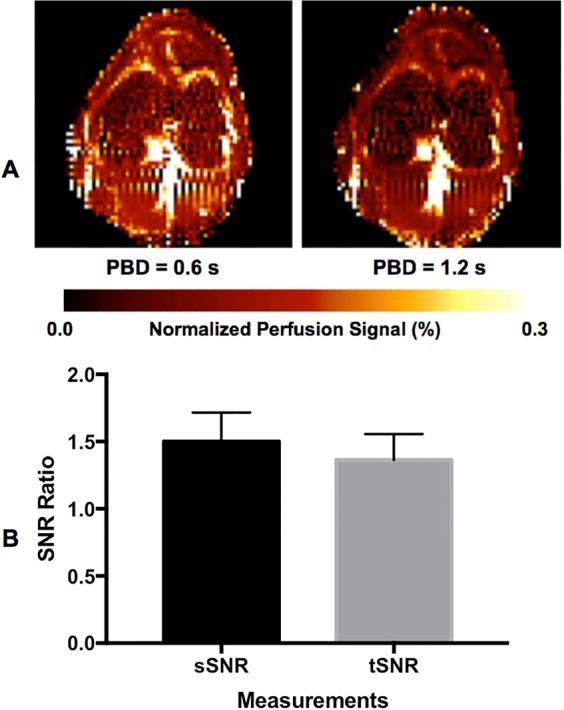
Perfusion-weighted images with two post-bolus delays (PBDs) from one participant (**A**) and ratios of spatial and temporal SNRs (sSNR and tSNR) measurements using two PBDs from five participants (**B**). Error bars represent standard deviations.

**Figure 2:**
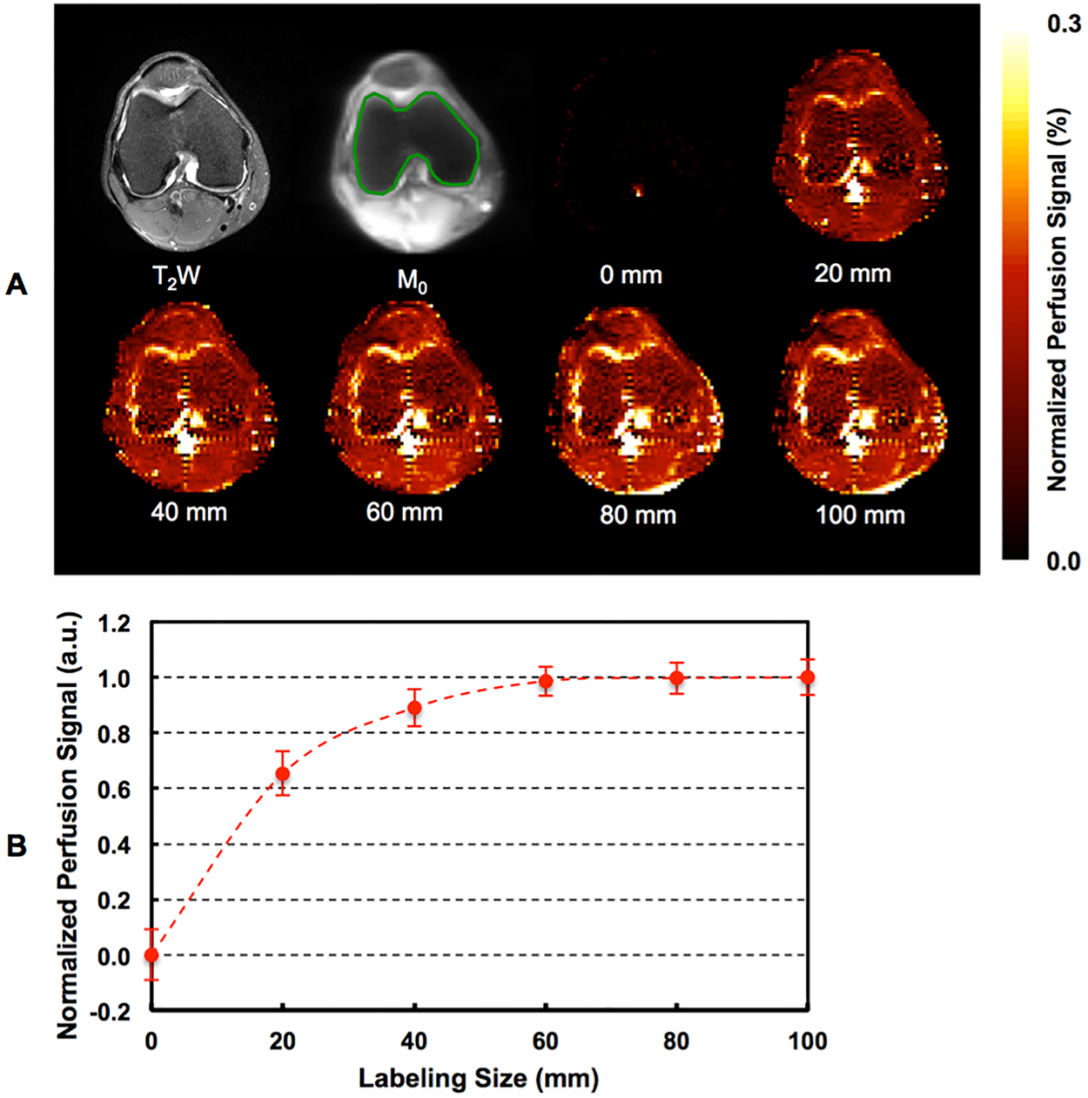
One typical participant’s anatomic T_2_-weighted image, M_0_ image with overlaid region of interest, perfusion-weighted images (**A**), and bone marrow perfusion signals (N = 3) (**B**) using different labeling sizes. Error bars represent standard deviations.

Knee bone marrow perfusion imaging was successfully performed with all volunteers. Results from the study with five participants using two post-bolus delay times (PBDs) suggest that the longer the PBD, the lower the bone marrow perfusion signal, and that the use of a longer PBD can help to minimize hyper-intensive intravascular signals within the bone marrow (Fig. 1). Study results from perfusion imaging using varied labeling sizes with three participants indicate that measured bone marrow perfusion signal changes with the size of ASL labeling (Fig. 2).

**Figure 3:**
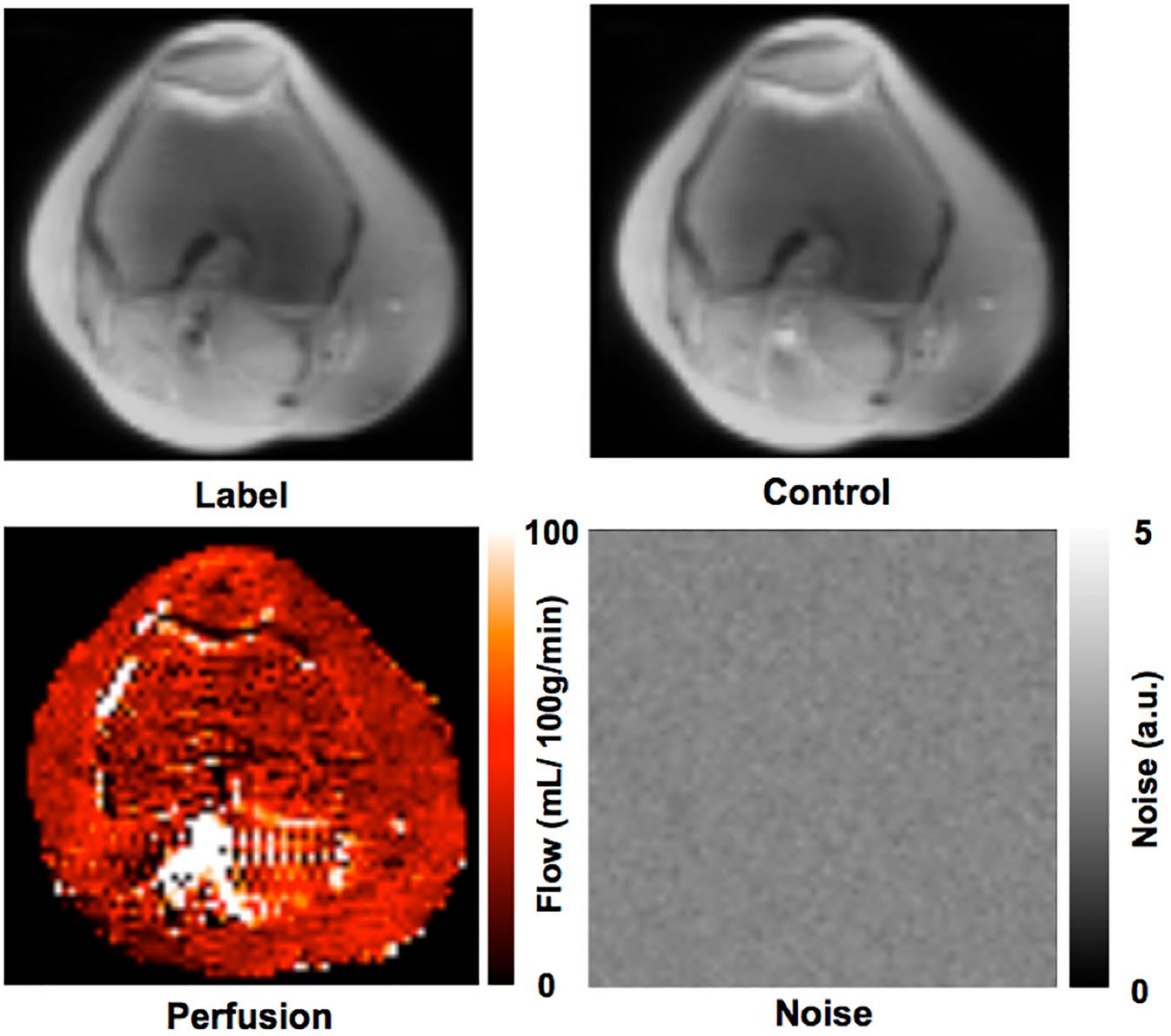
Label and control images and perfusion and noise maps for one participant.

**Figure 4:**
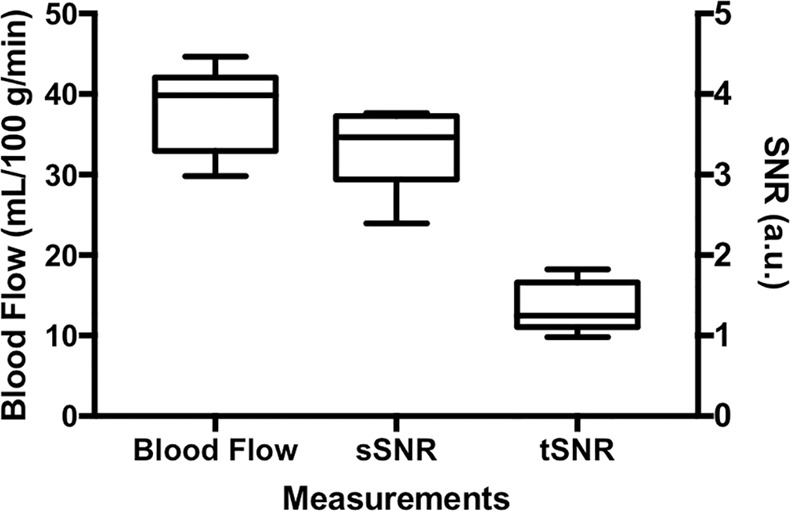
Bone marrow blood flow and perfusion signal spatial and temporal signal-to-noise ratios (sSNR and tSNR) from eight knees.

Quantitative bone marrow perfusion imaging results from the study using a 600 ms PBD are presented in Figs. 3 and 4: Fig. 3 shows label and control images and blood flow and noise maps from one representative participant; Fig. 4 presents a box and whiskers plot of bone marrow blood flow and spatial and temporal SNRs from eight participants. The means and standard deviations of bone marrow blood flow, spatial SNR, and temporal SNR were 38.3 ± 5.2 mL/100 g/min, 3.31 ± 0.48 and 1.33 ± 0.31, respectively.

**Figure 5:**
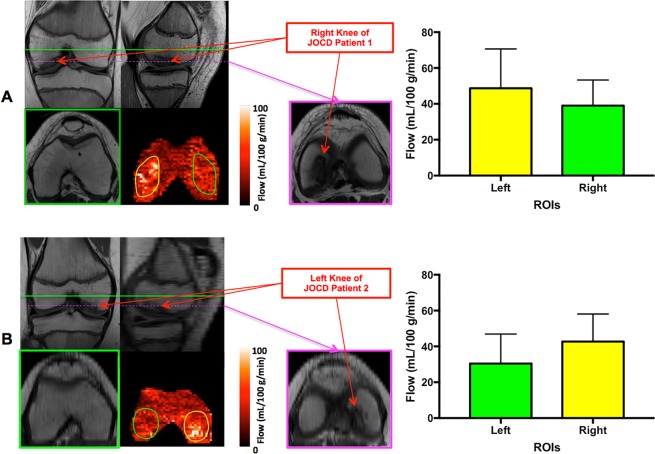
Proton-density-weighted TSE anatomic images, ASL perfusion maps and quantitative perfusion measurements within regions of interest (ROIs) from two JOCD patients in A and B, respectively. Green lines illustrate the slice positions for ASL perfusion imaging and corresponding anatomic images, and pink lines mark the slice positions for the distal anatomic images as shown on the right of perfusion maps. The JOCD lesions are indicated by red arrows. ROIs for the comparisons of bilateral perfusion levels in the knees, illustrated by yellow and green contours, have the same shapes and sizes. The observed hyper-perfusion signals are proximal to the JOCD lesions. Error bars represent standard deviations.

The imaging results from two JOCD patients are presented in Fig. 5. In the knees with diagnosed JOCD lesions, the blood flow measurements in ROIs proximal to the lesions were significantly higher than those in the same-size ROIs on the opposite sides of the knees: for both patients, p values from a two-tailed paired t test for perfusion values within two bilateral ROIs were less than 0.0001.

## Discussion

Bone perfusion imaging can provide new insights into underlying mechanisms of biological changes induced by injuries or diseases, help prognosticate skeletal diseases and injuries, and evaluate therapy response based on perfusion changes^1–3^. Due to its non-invasive and non-contrast-enhanced features, ASL imaging is an attractive and desired approach for the assessment of knee bone marrow perfusion. To our best knowledge, this is the first study that demonstrated the feasibility of knee bone marrow perfusion imaging using ASL at 3 T.

Our results show that knee bone marrow is not highly perfused with blood flow much lower than those in the brain and kidneys^20,23^. Despite low perfusion level in knee bone marrow, adequate perfusion SNR can be achieved by using a moderate image resolution (2 ×2 ×10 mm^3^) and a large number of perfusion signal averages (50 averages) (Fig. 4). In addition, our quantitative perfusion imaging study used a 600 ms PBD, instead of a 1200 ms PBD, to avoid excessive perfusion signal decay (Fig. 1). Although a longer PBD will allow labeled blood spins have more time to travel down the vascular tree into the capillary bed and minimize intravascular hyper-intensive signals within the bone marrow, this also results in a significant drop in perfusion image SNR and poor image quality. Furthermore, because of the slow blood flow in the knee area, even with a 1200 ms PBD, hyper-intensive signals were still observed around the edges and at the central regions of knee bone marrow area (Fig. 1A). For bone marrow perfusion quantification, to avoid potential undesired biases due to residual hyper-intensive signals and subtraction errors, trimmed mean signals within ROIs were used in the final perfusion signal measurements by excluding the 5% of voxels with the lowest and highest values. Such an approach can also help to reduce the impact of subtraction errors resulting from residual small motion^24^.

Importantly, our results show that the measured perfusion signal changes with the size of the ASL labeling region (Fig. 2), indicating that the knee bone marrow perfusion measurements are flow-associated. This is because the larger labeling size, the more labeled blood spins; and the more labeled blood spins, the higher the perfusion signal. Of course, these results also suggest that because of the slow blood flow around the knee, a labeling size larger than 60 mm may not further improve perfusion SNR.

Encouragingly, imaging results from JOCD patients suggest that ASL imaging may have the potential to detect bone marrow perfusion changes associated with the diagnosed lesions. As shown in Fig. 5, there exists hyper-perfusion in the regions proximal to the OCD lesions. Such hyper-perfusion may be due to revascularization or neovascularization associated with a healing process^1,25^.

PET imaging and DCE MRI have been applied for the assessment of bone perfusion in the past years. However, these imaging modalities can only provide indirect information about bone perfusion (e.g., 18F-Fluoride uptake in PET imaging or pharmacokinetic parameters in DCE MRI). Recently, studies have been performed to investigate the potential ability of ASL imaging in measuring bone marrow perfusion. One spine ASL imaging study at 3 T showed that vertebral bone marrow perfusion measurements from ASL imaging were correlated to pharmacokinetic parameters from DCE-MRI^21^. Motivated by the fact that 7 T can specifically benefit ASL imaging with increased perfusion SNR^23^, one exploratory study was performed at 7 T, suggesting that further technical development is needed to overcome technical challenges in order to improve the quality of ASL imaging and increase the reliability of knee bone marrow perfusion measurements^22^. Knee bone marrow perfusion measurements from this study are comparable to those measured at 7 T^22^, but lower than those in the spine^21^, which may be because bone marrow perfusion varies across different skeletal regions.

Effective and uniform fat suppression is critical for successful bone marrow ASL perfusion imaging. Knee bone marrow, unlike the brain and kidneys, is highly rich in fat. This makes fat suppression highly important to ensure label and control image signals mainly from water spins and minimize subtraction errors by reducing label and control image signal intensity. In addition, B_0_ inhomogeneity can adversely affect fat suppression across image space, resulting in non-uniform fat suppression and poor perfusion image quality. To minimize B_0_ inhomogeneity within imaging region for improved fat suppression, Siemens’ advanced B_0_ shimming was applied, which performs B_0_ shimming twice by using sequentially acquired B_0_ maps.

Increasing signal gain for the ASL image acquisition is also helpful for successful bone marrow ASL perfusion imaging. In our study, the signal dynamic range of ASL imaging was specifically adjusted to increase the signal intensity difference between fat-suppressed bone marrow tissue and the background. Although the use of a large signal dynamic range can result in signal saturation within the skin and muscles of the knee, it ensures that the signal level of fat-suppressed bone marrow tissue is larger than that of the background.

Our study has several limitations. First, the imaging coverage of the implemented ASL method is limited, only supporting a single slice acquisition. To overcome this limitation and better facilitate future research and clinical applications, it will be necessary to evaluate alternative image readout methods that are amenable to multi-slice ASL imaging and are insensitive to susceptibility-associated artifacts and distortion, such as Readout-Segmented Echo-Planar Imaging (RESOLVE)^26^. Second, in this proof of concept study, ASL imaging parameters (e.g., PBDs) were empirically chosen with young subjects. For studies with other populations, such as the elderly, ASL parameters need to be further verified or optimized, and knee bone marrow perfusion values can also vary across different populations. Third, no background suppression^27^ was applied for our knee bone marrow ASL studies. Due to imperfect inversion, the application of background suppression can cause loss of perfusion signal that is already low in knee bone marrow. The potential benefits of background suppression can be assessed in the future. Forth, although our results have shown that ASL measurements within knee bone marrow are flow-associated, further validation studies are needed, such as investigating the correlations between blood flow measurements from ASL imaging and pharmacokinetic parameters from DCE MRI. Finally, although the potential ability of ASL imaging to detect disease-associated bone marrow perfusion changes has been demonstrated with two JOCD patients, these results should be interpreted cautiously. Both the direction (hyper-perfusion or hypoperfusion) and the origins of disease-associated perfusion changes need to be further investigated with more JOCD patients in the future. In addition, more studies should be performed to explore the prognostic value of bone marrow ASL imaging, such as whether the detected perfusion changes are associated with patient outcome.

In summary, this study demonstrates that it is feasible to perform ASL imaging of knee bone marrow in the distal femoral condyle at 3 T.

## Materials and Methods

### Participants

Eight young volunteers with no history of knee diseases or injuries (three males and five females, 26 ± 2 years old, mean ± standard deviation (S.D.)) participated this study. To investigate the potential ability of ASL imaging to detect disease-associated bone marrow perfusion changes, two male patients diagnosed with JOCD were also recruited for knee ASL imaging: one 15 year-old patient with JOCD in the right knee and another 11 year-old patient with JOCD in the left knee. These lesions were diagnosed as stage II according to the classification system proposed by Ellermann *et al*.^28^. This study was approved by the ethics committee of the University of Minnesota, and performed in accordance with the institutional guidelines and regulations for human research. Prior to imaging study, written informed consent was obtained from each of the participants and the legal guardian of the JOCD patient after having full understanding of the study.

### MRI

Knee imaging studies were performed on a Siemens 3 T MRI scanner (Siemens Healthcare, Erlangen, Germany) with a 15-channel receive, 1-channel transmit knee coil (QED, Mayfield Village, OH). The imaging protocol included scout localizer, high-resolution anatomic imaging scans using T_2_-weighted and proton-density-weighted turbo spin echo (TSE) sequences, and knee bone marrow perfusion imaging.

**Figure 6:**
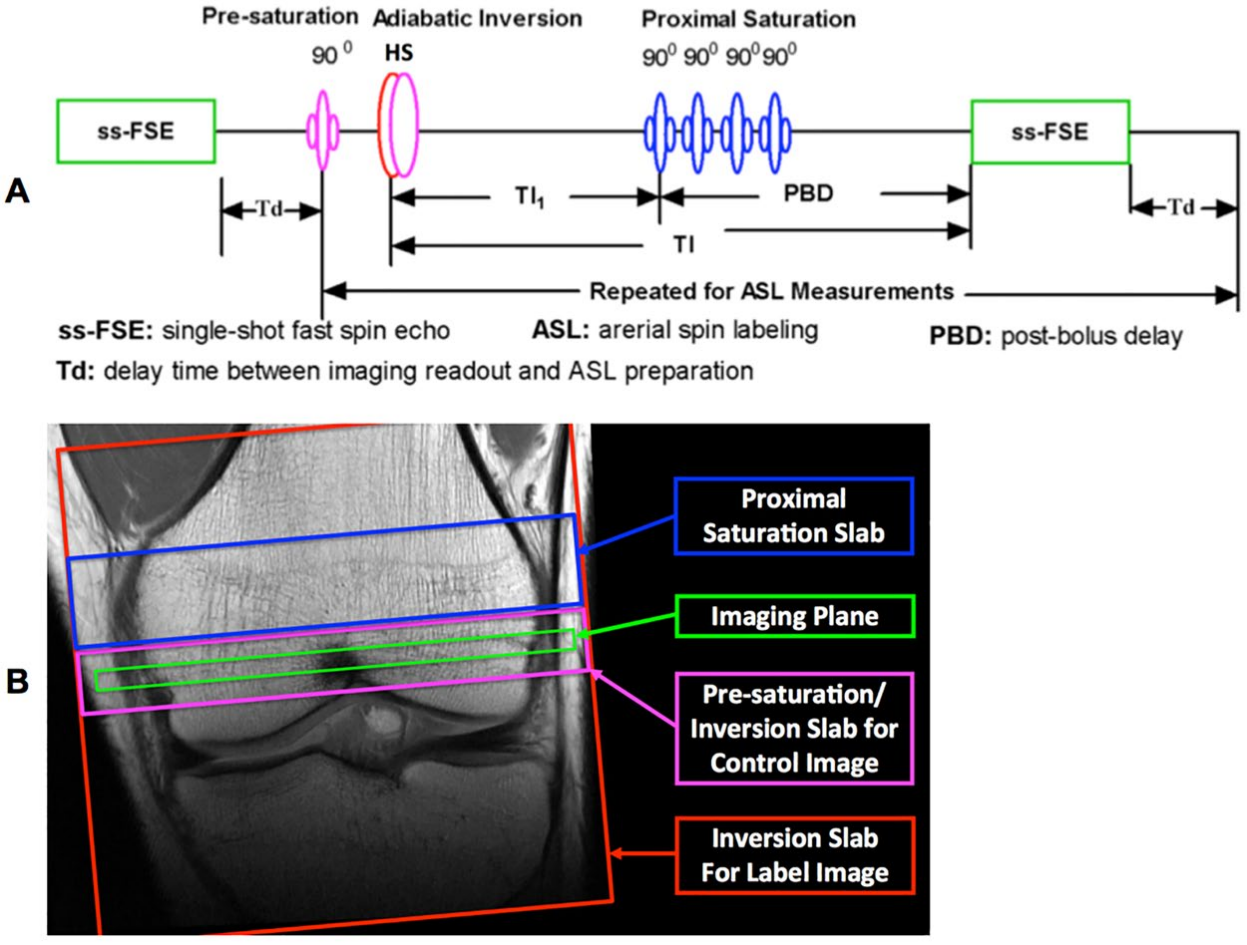
Sequence diagram (**A**) and RF pulse spatial positions at the distal femoral condyle (**B**) of the FAIR ss-FSE method. A single ss-FSE image is first acquired to measure fully-relaxed bone marrow magnetization (called M_0_ image) before perfusion image acquisitions. This sequence consists of a pre-saturation RF pulse at the slice location, adiabatic inversion RF pulses for either label or control images, and four proximal saturation RF pulses to define the temporal bolus width (TI_1_) followed by a post-bolus delay (PBD) (TI-TI_1_).

ASL perfusion imaging utilized a FLow-sensitive Alternating Inversion Recovery (FAIR) method^29^ for labeling and a single-shot fast spin echo (ss-FSE) sequence for perfusion image readout (referred to as FAIR ss-FSE) to avoid severe susceptibility-associated image distortion and ghosting artifacts usually observed in EPI^22^. The sequence diagram and relative spatial locations of each RF pulse in the sequence are illustrated in Fig. 6. In this sequence, a single ss-FSE image is first acquired to measure the fully relaxed renal tissue magnetization (referred to as the M_0_ image) prior to the acquisition of label and control images. The ASL preparation module consists of: a pre-saturation RF pulse applied at the location of the imaging slice; an optimized HS4 adiabatic pulse that alternates between a small inversion slab for control images and a large inversion slab for label images; a specified delay time (TI_1_ in Fig. 6A) after the inversion RF pulse to define the temporal bolus width of the labeled spins; saturation RF pulses targeting a slab proximal and parallel to the imaging slice; and a post-bolus delay (PBD = TI-TI_1_, Fig. 6A) to allow the labeled blood to travel down the vascular tree into the small arterioles and minimize undesired hyper-intense intravascular signals. To help ensure label and control image signals within the bone marrow were dominantly coming from water spins, the ss-FSE image acquisition employed fat saturation. To boost the water signal level in the bone marrow, the signal gain was elevated for image data acquisition.

To minimize B_0_ inhomogeneity within the imaging region and improve fat saturation, Siemens’ advanced B_0_ shimming was applied. Siemens’ advanced B_0_ shimming method performs B_0_ shimming twice by using sequentially acquired B_0_ maps. To overcome B_1_^+^ inhomogeneity and ensure the adiabatic condition is met for the inversion RF pulse throughout the entire labeling region at the proximal side of the imaging slice, a nominal flip angle at least 1.5 times that needed for adiabatic inversion was applied for the HS4 inversion RF pulse with a 16 ms duration and a time-bandwidth product of 20^30^.

Knee bone marrow perfusion imaging was performed with a single oblique transverse slice as shown in Fig. 6B. To ensure at least ¾ coverage of the knee coil over the labeling region, the knees of the participants were positioned with the proximal aspect of the patella positioned at the center of the coil. To avoid ghosting artifacts due to blood flow in large vessels, the left-to-right phase-encoding direction was oriented perpendicular to the anterior-posterior direction of the knee.

Parameters for quantitative ASL knee bone marrow perfusion imaging were as follows: TR/TE = 3000/13 ms, flip angle = 90°, FOV = 140 × 140 mm^2^, matrix size = 70 × 70, in-plane resolution = 2 × 2 mm^2^, phase encoding direction = left to right, slice thickness = 10 mm, partial Fourier = 5/8, GRAPPA parallel acceleration factor (R) = 2 with 24 separately acquired reference lines, labeling time (TI_1_)/total delay time (TI) = 600/1200 ms; control/labeling inversion slab size = 30/230 mm; number of label and control images = 100; slab thickness/RF duration/interval of proximal saturation = 100 mm/25 ms/50 ms; and total imaging time = ~5 minutes. For each ASL scan, 200 noise images were acquired after the perfusion acquisition by turning off RF pulses to facilitate perfusion SNR analysis^20^.

To evaluate if the measurements of knee bone marrow ASL imaging are flow-associated, perfusion studies were performed with three volunteers using the same imaging parameters as described above except with application of varied labeling sizes: 0, 20, 40, 60 80, and 100 mm. For five participants, perfusion imaging using a 1200 ms post-bolus delay (PBD) was also performed to evaluate how an increase in total delay time affects the hyper-intense intravascular artifacts and spatial and temporal perfusion SNRs.

### Image processing and data analysis

For each ASL series, 2D motion correction was performed using FSL toolbox (FMRIB, Oxford, UK)^31^. After motion correction, label and control images were pair-wise subtracted to obtain perfusion-weighted images. The perfusion-weighted images were subsequently averaged to get a mean perfusion-weighted image that was used for perfusion quantification. The calculation of the blood flow map and region of interest (ROI) analyses were performed using in-house scripts implemented in Matlab 8.6 (MathWorks, Natick, MA, USA).

Blood flow quantification utilized the single compartment blood flow quantification model^32^:

1 $${\rm{BMBF}}({\rm{r}})=\Delta {\rm{M}}({\rm{r}})/[2{\rm{\alpha }}\times {{\rm{M}}}_{0}({\rm{r}})\times {{\rm{TI}}}_{1}\times \exp (-{\rm{TI}}/{{\rm{T}}}_{{\rm{1b}}})]$$

where r is the spatial location of imaging voxel, ΔM the perfusion signal from a mean perfusion-weighted image, M_0_ the fully relaxed magnetization of bone marrow, TI_1_ the sequence-defined temporal bolus width, TI the total delay time, T_1b_ the longitudinal relaxation time of the arterial blood, 1660 ms^33^, and α the labeling efficiency, 0.95^30^.

Spatial and temporal SNR analyses were performed for perfusion signals within the bone marrow. The spatial noise map was estimated as the standard deviation map of 200 noise images^20^. The spatial SNR map was then estiamted as follows:

2 $${{\rm{S}}{\rm{N}}{\rm{R}}}_{{\rm{s}}{\rm{p}}{\rm{a}}{\rm{t}}{\rm{i}}{\rm{a}}{\rm{l}}}({\rm{r}})={\rm{S}}({\rm{r}})\times \sqrt{({{\rm{N}}}_{{\rm{a}}{\rm{v}}{\rm{g}}}/2)}/\sigma ({\rm{r}})$$

where *S(r)* represents perfusion signals, *N*_*avg*_ the number of temporal perfusion signal averages, and σ(r) the spatial noise. The temporal SNR map was calculated as the ratio of the mean perfusion-weighted image to the temporal standard deviation of all perfusion-weighted images.

ROIs were conservatively defined on M_0_ images to cover the bone marrow as illustrated in Fig. 2. Trimmed mean signals within ROIs were used in the final perfusion signal measurements by excluding the 5% of voxels with the lowest and highest values^24^.

Statistical analyses were performed using the GraphPad Prism (GraphPad Software, La Jolla, CA) software. Statistical significance was defined as a *p* < 0.05.

## Data Availability

The majority of data generated or analyzed during this study are included in this article. Additional information is available from the corresponding author on reasonable request.
